# The psychology of digital credit: the association of financial anxiety and impulsive traits with “buy now, pay later” delinquency

**DOI:** 10.3389/fpsyg.2026.1950938

**Published:** 2026-10-12

**Authors:** Chenchen Ma, Mei Ge, Mingqiu Gu, Ziran Ding

**Affiliations:** 1 School of Economics and Finance, Xi'an Jiaotong University, Xian, China; 2 Faculty of Business, City University of Macau, Macao, Macao SAR, China; 3 Commerce (accounting), University of New South Wales, Sydney, NSW, Australia; 4 Faculty of Business, Zhuhai College of Science and Technology, Zhuhai, China

**Keywords:** buy now pay later, consumer psychology, financial anxiety, impulsive buying tendency, objective financial literacy

## Abstract

**Introduction:**

Digital credit structures, exemplified by the “Buy Now, Pay Later” (BNPL) model, combine frictionless transactional accessibility with the underlying psychological vulnerabilities of users. This cross-sectional investigation maps the statistical covariation between perceived financial anxiety and distinct digital credit usage dimensions.

**Methods:**

Utilizing a dataset of 890 respondents (708 of whom are active BNPL users), regression and mediation analyses was conducted. The study evaluated subjective financial anxiety, BNPL usage (frequency, dependence, hedonic preference, and delinquency), impulsive buying tendency, and objective financial literacy.

**Results:**

Analyses reveal an absence of significant correlation linking financial anxiety to operational usage frequency or overarching bill dependence. However, a robust positive covariation emerges between heightened financial anxiety and both the allocation of credit toward non-essential hedonic consumption and the propensity for debt delinquency. Impulsive buying tendency acts as a stable trait that shows a statistical indirect association in this cross-sectional relationship, and objective financial literacy does not significantly moderate this association.

**Discussion:**

The structural pattern characterizes maladaptive digital default primarily as an emotion-regulation phenomenon—associated with the convergence of subjective insecurity and impulsive traits—rather than a mere deficit in mathematical knowledge. This highlights the need to incorporate behavioral friction elements into fintech interfaces.

## 1 Introduction

Globally shifting consumer markets feature payment evolutions that correspond with differentiated individual financial decision-making patterns. Digital credit arrangements, notably the BNPL framework, circumvent conventional entry barriers associated with traditional credit systems, embedding installment mechanisms into diverse consumption environments. While providing transactional utility and contributing to the broader expansion of digital financial inclusion (Xia et al., 2025), these structures demonstrate a statistical relationship with users' psychological insecurities (Aalders, 2023; Valenciano et al., 2025). Academic inquiries into digital credit have historically concentrated on macroeconomic financial inclusion, leaving a conceptual gap regarding the intersection of these immediate payment environments with individual psychological vulnerabilities. To address this gap, it becomes essential to move beyond merely tracking generalized digital credit adoption, and instead explicitly distinguish routine usage intensity from psychologically maladaptive credit behaviors.

Economic cycle variations and structural labor realignments often correspond with varying degrees of psychological tension among consumers confronting income uncertainty. Financial anxiety, acting as a psychological trait specifically tethered to personal economic status, provides a metric for interpreting modern consumption distributions (An et al., 2025). Traditional life-cycle hypotheses posit that rational economic agents, upon perceiving financial constraint, will uniformly orient toward contractionary habits to accumulate precautionary savings. Psychological observations detail a contrasting pattern: worries regarding personal financial standing do not uniformly correspond with conservative coping strategies but frequently co-occur with continuous, logic-divergent credit consumption (Coffey et al., 2024). This divergence between theoretical assumptions and observable actions necessitates an examination of the correlational properties shared by these modern phenomena.

The present study approaches BNPL as a behavioral vessel carrying distinct emotional and cognitive variables, moving beyond its categorization as a functional economic tool. The central analysis explores whether reported financial pressure correlates positively with specific maladaptive patterns—including frequent application, elevated reliance, and credit delinquency—and whether impulsive buying traits, functioning as proxies for inhibitory control variance, map onto this structural network via an indirect statistical association. The potential moderating capacity of an individual's objective financial literacy is simultaneously integrated into the evaluation matrix.

Theoretical bridges are constructed between behavioral finance and personality psychology through the integration of the scarcity mindset framework and the ego depletion concept. The nexus of financial anxiety and credit reliance aligns well with interpretations centered on human self-regulation (Baumeister et al., 2007). Subjective financial distress transcends mathematical budget boundaries, frequently co-occurring with self-directed critiques regarding economic status, a posture relating to threat-associated physiological states (Gilbert, 2009). Individuals often resort to compensatory mechanisms, such as impromptu acquisitions via accessible BNPL interfaces, in pursuit of temporary emotional regulation (Heatherton and Wagner, 2011). Under this lens, digital overdrawing is conceptualized as an immediate emotional coping manifestation associated with constrained cognitive bandwidth. Employing a quantitative statistical control approach, this research assesses the net correlations connecting emotional variables to non-payment behaviors, aiming to refine the descriptive limits of behavioral finance literature.

## 2 Literature review and research hypotheses

### 2.1 Connotation and behavioral characteristics of “buy now, pay later” usage patterns

Functioning as a short-term financing alternative available at retail checkouts, BNPL bypasses the underwriting procedures characteristic of credit card systems. Consumer psychology research examining the adoption of these platforms primarily notes a relationship with a diminished psychological threshold regarding payment execution (Di Maggio et al., 2022). Confronting pending bills, individuals encountering installment options register altered immediate price perceptions, a cognitive pattern demonstrating observable positive covariation with expanded overall expenditure volumes. Current academic perspectives increasingly delineate BNPL adoption as a continuous multidimensional behavioral spectrum, pivoting away from earlier binary categorizations of mere adoption versus non-adoption (Cook et al., 2023; Guttman-Kenney et al., 2023). Given that this digital credit behavior occurs within a rapidly evolving domestic financial environment characterized by the deep integration of digital payment infrastructures into daily consumption patterns (Jiang and Yuan, 2026), deconstructing this behavioral pattern involves metrics encompassing application frequency, proportional bill dependence, and documented repayment issues. Research by Gerrans et al. (2022) indicates that populations relying frequently on digital credit present financial characteristics markedly divergent from their low-frequency counterparts. The contextual application of this digital tool is also exhibiting structural reconfigurations. While a demographic segment utilizes BNPL to maintain baseline daily utility, another demographic increasingly applies these instruments toward non-essential hedonic categories, including entertainment electronics and leisure services (Foster et al., 2022; Jain and Raman, 2023; Kumar and Nayak, 2024). This heterogeneous classification of end-use environments offers analytical dimensions for identifying related psychological variables.

### 2.2 Relationship between financial anxiety and BNPL usage patterns

Financial anxiety represents a persistent state of subjective apprehension documented among individuals assessing their existing obligations, anticipated income streams, or immediate liabilities (Archuleta et al., 2013; Mamani-Benito et al., 2025; Shah et al., 2025). Shapiro and Burchell (2012) outline that this condition remains distinct from generalized clinical anxiety, operating instead as a highly contextualized psychological variable. Placing this trait within the ecosystem of digital credit, Scarcity Mindset Theory delivers an applicable descriptive framework. Studies led by Mullainathan and Shafir (2013) suggest that the cognitive bandwidth available to individuals confronting ongoing pressure is disproportionately allocated toward immediate requisites. This reallocation correlates with a heightened valuation of immediate acquisition, running parallel to a discounted weighting of subsequent obligations.

Recent evidence from consumer psychology highlights that negative affective states can systematically alter consumer evaluations and decision-making logic (Yang et al., 2026). Furthermore, psychological distress has been empirically linked to divergent patterns of technology-mediated behavior and maladaptive digital responses (Chu et al., 2025; Donou-Adonsou and Leslie-Piper, 2026). Cross-sectional compilations indicate that individuals returning higher financial anxiety scores report corresponding difficulties preserving basic cash flow equilibrium. Managing immediate financial demands, populations registering elevated stress markers display an increased statistical probability of integrating digital credit within their purchasing routines, leading to the following foundational hypothesis: Hypothesis H1: Identified financial anxiety levels correlate positively with the frequency of BNPL application and corresponding proportional bill dependence.

Repayment adherence constitutes a core metric for determining individual credit health. Chronic financial tension routinely co-occurs with compromised executive planning capabilities. Lusardi and Tufano (2015) documented that demographics experiencing heightened subjective stress present increased incidences of debt accumulation. Translated to the BNPL context, this relationship surfaces through late fee accumulation and recurring installment modifications. Hypothesis H2: Consumers characterized by elevated financial anxiety demonstrate a related propensity for encountering delinquency or requiring obligation extensions when engaging with BNPL services.

Evaluating categorized fund allocations provides insight into the behavioral logic anchoring digital credit habits. Drawing on Compensatory Consumption Theory (Rucker and Galinsky, 2008), individuals navigating psychological strain display patterns of acquiring previously unneeded goods, attempting to secure immediate emotional equilibrium. When unencumbered payment platforms offer leverage surpassing immediate liquidity, this accompanying compensatory purchasing pattern routinely expands beyond staple items, aligning with hedonic sectors capable of delivering instantaneous stimulation. Hypothesis H3: Measured individual financial anxiety positively correlates with the application of BNPL tools toward non-essential hedonic consumption scenarios.

### 2.3 The mediating mechanism of impulsive buying tendency

Clarifying the indirect correlational pathways linking financial anxiety to credit patterns necessitates a focus on individual consumer trait distributions. Impulsive buying tendency, operating as a relatively stable dispositional marker, captures an individual's likelihood of executing acquisitions synchronized with immediate emotional fluctuations, absent thorough deliberation (Le, 2025; Nusir et al., 2026; Rook and Fisher, 1995). Standard cognitive models require individuals engaging in long-term financial planning to suppress instantaneous acquisition desires. The Ego Depletion paradigm (Baumeister et al., 1998) alternatively posits that personal self-control reserves operate within limited capacities.

It is crucial to theoretically distinguish between trait-level impulsivity (a relatively stable personality disposition) and state-level ego depletion (a temporary exhaustion of self-regulatory resources). We theoretically posit that respondents managing elevated financial anxiety dedicate substantial mental capital toward processing expected bills and future uncertainty. This prolonged psychological load acts as a severe environmental stressor, precipitating a state of chronic ego depletion. It must be explicitly acknowledged that ego depletion serves as a theoretical explanation for the observed behavior, rather than a mechanism empirically tested in this particular study. In other words, systemic emotional distress is statistically associated with higher impulsive buying tendency, which in turn is associated with delinquency (Hilmi and Pratika, 2021; Weun et al., 1998). This indirect path manifests in cross-sectional metrics as instances of budget allocation collapse, utilizing readily available BNPL architectures as conduits for these exhausted decision-making processes. Compared to standard usage frequency, actual delinquency events more directly capture this breakdown of personal financial perimeters. The study explores this structural dynamic by examining the intermediary role occupied by amplified impulsive traits within the identified dataset. Hypothesis H4: Impulsive buying tendency exhibits an indirect statistical association between financial anxiety and recognized delinquency markers in BNPL applications.

### 2.4 Potential moderating mechanism of objective financial literacy

Contemporary financial observation would lose its intervention value if all individuals registering anxiety uniformly engaged in impulsivity and subsequent default. Heterogeneity regarding financial risk management is theoretically tied to cumulative objective financial knowledge, treated as a proxy variable for cognitive processing abilities (Aristei and Gallo, 2021; Lusardi and Mitchell, 2014). Grounded in the conventions set by Lusardi and Mitchell (2011), objective financial literacy captures a respondent's accurate comprehension regarding compound interest application, currency inflation concepts, and asset diversification logic.

Within credit performance analyses, this knowledge base operationalizes as an interacting cognitive variable (Abdallah et al., 2025). Prior texts suggest populations maintaining robust theoretical knowledge frameworks navigate implicit interest rates and late fee matrices with enhanced calculation precision, mapping onto more calculated debt acquisition postures. Among segments returning lower baseline knowledge scores, the absence of cognitive anchoring potentially amplifies the relationship between impulsive acquisition tendencies and ultimate bill crises (Ainy et al., 2025; Khandelwal et al., 2022). This structural premise initiates an inquiry from a dual-process viewpoint, examining if factual knowledge scores demonstrate the capacity to interact with the pathway linking emotional impulsivity to non-payment. Hypothesis H5: Objective financial literacy presents measurable interactive characteristics within the statistical relationship between impulsive buying tendencies and BNPL delinquency; namely, among populations with higher literacy scores, the positive correlation connecting impulsive traits to default events presents at a reduced magnitude.

Correlating these theoretical deductions, the study structures a model outlining the associations linking financial anxiety to specific digital credit usage topographies. Model composition detailing these hypothetical pathways is outlined in Figure 1.

**Figure 1:**
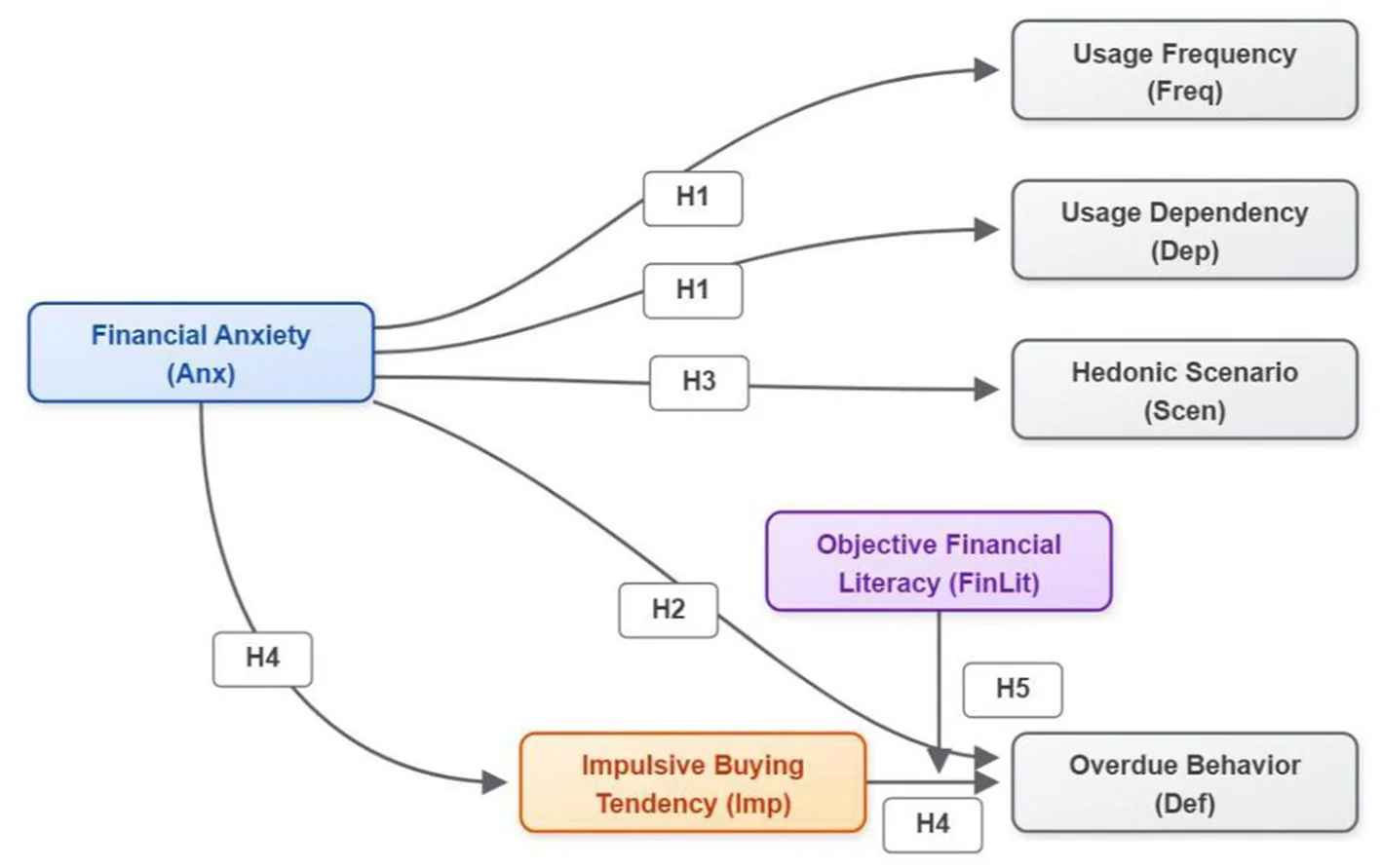
Research hypotheses and theoretical correlation mechanism model. Operating within the ego depletion framework, Impulsive Buying Tendency functions as a trait-level vulnerability marker for self-regulatory lapse, with Objective Financial Literacy conceptualized as a cognitive processing proxy.

## 3 Research methodology

### 3.1 Sample and data collection

The underlying dataset originates from a targeted, single-point cross-sectional survey distributed across a recognized online survey platform. Deployment occurred during a period devoid of major e-commerce promotional operations, attempting to isolate baseline consumption patterns from external marketing interference. Questionnaire construction followed standard protocols concerning voluntary participation and informed consent integration. The research design incorporated response duration parameters and embedded attention verification items to filter perfunctory submissions.

The initialization phase yielded 950 raw survey responses. Preparation for formal statistical processing involved comprehensive data cleaning protocols, prioritizing the following exclusion criteria: rejection of informed consent directives; response durations failing to meet minimum established thresholds; inaccurate responses on attention verification questions; identifiable mechanical response patterns; and missing data concentrated within core variable blocks. Psychological scale score outliers were identified and isolated utilizing *Z*-scores. This filtration returned a final cohort of 890 valid questionnaires (retention rate: 93.68%, see Supplementary Data Sheet 1). Applying fundamental screening criteria regarding digital credit application history, this pool was subsequently segmented into an active analysis group (*n* = 708) alongside a non-using reference group (*n* = 182).

Demographic classification presents a cross-sectional distribution mapping onto established profiles of contemporary digital credit consumers (Table 1). Gender representation remains structurally balanced. Age demographics heavily feature younger adult cohorts spanning 18–30 years (aggregating 63.37% of the sample) (Izham et al., 2025; Mappadang et al., 2025). Educational attainment predominantly clusters at the undergraduate baccalaureate level or higher. These sample metrics align with market landscapes where retail financial instruments maintain broad infiltration among younger, digitally integrated populations addressing routine transaction protocols.

**Table 1:** Distribution of sample demographic characteristics (*N* = 890).

Variable category	Classification dimension	Frequency (*n*)	Percentage (%)	Cumulative percentage (%)
Gender (Q2)	Male	422	47.42	47.42
	Female	446	50.11	97.53
	Prefer not to disclose	22	2.47	100.00
Age group (Q3)	18–25 years old	298	33.48	33.48
	26–30 years old	266	29.89	63.37
	31–40 years old	194	21.80	85.17
	41–50 years old	87	9.78	94.95
	50 years old and above	45	5.06	100.00
Highest education (Q4)	High school/technical school and below	82	9.21	9.21
	Junior college/vocational college	228	25.62	34.83
	Undergraduate	446	50.11	84.94
	Master's and above	134	15.06	100.00
Occupational status (Q5)	Full-time student	213	23.93	23.93
	Full-time job	492	55.28	79.21
	Freelance/flexible employment	136	15.28	94.49
	Unemployed/out of work	49	5.51	100.00
Monthly income level (Q6)	Below 3,000 RMB	187	21.01	21.01
	3,000–5,000 RMB	226	25.39	46.40
	5,001–10,000 RMB	265	29.78	76.18
	10,001–20,000 RMB	127	14.27	90.45
	Above 20,000 RMB	85	9.55	100.00

### 3.2 Variable measurement and operationalization

Translating latent psychological concepts into assessable empirical indicators, the investigation unpacks the dependent macro-variable “digital credit usage patterns” into four cross-sectional metrics: usage frequency (Freq), usage dependence (Dep), consumption scenarios (Scen), and delinquency performance (Def).

Usage frequency (Freq) and dependence (Dep) correspond to scaled self-reports detailing raw application rates and the proportion of total monthly expenditure routed through credit instruments. Enhancing explanatory cohesion, the options were standardized directionally; ascending numerical scores strictly reflect increasing physical frequency and heightened payment-channel dominance.

Consumption scenarios (Scen) and delinquency performance (Def) are coded based on the heterogeneity of item classification and repayment adherence. Scenarios are bisected into essential vs. hedonic categories based on respondent selections, isolating emotion-associated compensatory activity. Repayment performance operates on a four-point sequential scale reflecting the operational status of intertemporal obligations.

Financial anxiety (Anx), serving as the primary independent variable, is evaluated via the Financial Anxiety Scale (FAS) (Archuleta et al., 2013). This instrument applies a 5-point Likert configuration capturing individualized economic stress. The mediating variable, impulsive buying tendency (Imp), applies the six-item Impulse Buying Tendency Scale (IBTS) (Weun et al., 1998) to scale individual propensity regarding the suppression of sudden acquisition decisions.

Objective financial literacy (FinLit) is measured utilizing a cumulative correct-answer summation derived from standard baseline assessments (Lusardi and Mitchell, 2011). The three-item module tests comprehension of compound interest, inflationary shifts, and risk dispersion, with scores bounded between 0 and 3 points. Specifically, respondents were evaluated on their ability to calculate a 2% compound interest over 5 years, assess purchasing power under concurrent 1% interest and 2% inflation rates, and correctly identify the comparative risk between single company stocks and mutual funds. Although a concise three-item measure possesses inherent limitations regarding exhaustive construct coverage, this specific module is employed as it constitutes a universally validated baseline for capturing fundamental mathematical-economic rationality (Lusardi and Mitchell, 2014). Within the active user cohort (*n* = 708), the correct response rates—functioning as explicit item-level difficulty metrics—were 63.1% for the compound interest module, 53.0% for the inflation assessment, and 48.4% for the risk dispersion item. These stratified difficulty levels confirm the survey's capacity to capture varying degrees of cognitive financial proficiency. Furthermore, this foundational metric explicitly targets general objective financial knowledge; it remains conceptually distinct from domain-specific capabilities, such as debt literacy or digital financial literacy, which are increasingly relevant given that BNPL behaviors manifest within complex digital payment ecosystems. The operational definitions, along with the baseline descriptive statistics of these focal continuous constructs for the active user cohort, are summarized in Table 2.

**Table 2:** Operational definitions and basic descriptive statistics of core continuous variables (*n* = 708, user group only).

Variable name	Symbol	Measurement source	Number of items	Mean (*SD*)	Minimum	Maximum
Financial anxiety	Anx	Q12–Q17	6	3.13 (0.84)	1.00	5.00
Impulsive buying tendency	Imp	Q18–Q23	6	3.08 (0.83)	1.00	5.00
Usage frequency	Freq	Q8	1	2.76 (0.83)	1.00	4.00
Usage dependence	Dep	Q9	1	2.32 (0.88)	1.00	4.00
Objective financial literacy	FinLit	Q24–Q26	3	1.65 (0.94)	0.00	3.00

### 3.3 Model specification

Addressing the distinct distribution properties of the multi-dimensional dataset, the study structures corresponding statistical models to track the covariation network linking financial anxiety to behavioral markers:


$$\begin{array}{l} Y_{i}=\alpha +\beta_{1}Anx_{i}+\gamma X_{i}+\epsilon_{i} \end{array}$$

In Equation 1, *Y*_*i*_ operates as the placeholder for ordered variables, specifically usage frequency (Freq) and usage dependence (Dep). While ordered choice models are structurally applicable to stepped responses, the current sample dimension and the progressive linear trend observed in the current cross-section support the application of Ordinary Least Squares (OLS) for primary estimation.

Addressing the binary variable tracking non-essential hedonic scenarios (Scen), the equation employs Binary Logistic Regression protocols:


$$\begin{array}{l} \ln\left(\frac{P_{i}}{1-P_{i}}\right)=\alpha +\beta_{1}Anx_{i}+\gamma X_{i}+\epsilon_{i} \end{array}$$

In Equation 2, *P*_*i*_ represents the predicted probability of the respondent applying credit mechanisms toward hedonic allocations.

Delinquency (Def) modeling retains baseline OLS structures to facilitate the calculation of determination coefficient margins (Δ*R*^2^) within hierarchical steps, ensuring conformity with subsequent path decomposition algorithms. However, because BNPL delinquency operates as an ordered four-category outcome, supplementary ordered logistic regression models were simultaneously specified in the robustness phase to verify that substantive conclusions remain unchanged despite estimator differences.

Moving to structural pathway assessments, the investigation incorporates the PROCESS macro platform developed by Hayes (2017). After establishing foundational demographic controls, Model 4 processes non-parametric Bootstrap resampling computations (5,000 internal replications) examining the indirect structural association connecting financial anxiety variables, impulsive buying markers, and final delinquency outcomes (Equation 3):


$$\begin{array}{l} Def_{i}=c^{\prime }Anx_{i}+bImp_{i}+\gamma X_{i}+\epsilon_{i} \end{array}$$

Within the boundary condition testing phase, the inquiry utilizes Model 14 to introduce objective financial knowledge scores (FinLit) as an interacting term on the described mediation path. This step statistically evaluates if correlation strengths linking impulsivity and delinquency register variability conditioned upon individual cognitive capacities. Independent terms involved in interaction products underwent standard mean-centering preprocessing. Multi-Group Structural Equation Modeling (Multi-Group SEM) protocols were additionally applied to verify the structural equivalence of these covariation dynamics across varying demographic segments.

## 4 Results analysis

### 4.1 Reliability, validity, and common method bias tests

To mitigate potential common method bias (CMB) at the research design stage, procedural controls were actively implemented during data collection. Specifically, the survey guaranteed strict response anonymity and explicitly informed participants that data would be utilized exclusively for aggregate academic purposes, thereby minimizing evaluation apprehension and social desirability tendencies. Confirming alignment between theoretical constructs and empirical items acts as a prerequisite prior to exploring specific covariations. Initial consistency measures document Cronbach's α statistics of 0.869 relative to the subjective financial anxiety construct and 0.829 regarding impulsive buying measurements. Corresponding composite reliability (CR) metrics register at 0.870 and 0.830, occupying values above universal social science benchmarks (0.70). The exploratory factor module documents a KMO test statistic of 0.892, with Bartlett's sphericity testing rejecting the baseline proposition mapping zero correlation between the variables (χ^2^ = 3948.991, *df* = 66, *p* < 0.001).

Confirmatory Factor Analysis (CFA) further documents the independence of the two-factor framework. The measurement model yielded excellent goodness-of-fit ratios: χ^2^/*df* = 36.214/53 = 0.683, CFI = 1.000, TLI = 1.000, RMSEA = 0.000, GFI = 0.991, AGFI = 0.989, reflecting a highly distinct and concisely structured two-factor estimation.

Table 3 details that the financial anxiety sub-items present standardized loadings between 0.663 and 0.767 against the latent factor, alongside an average variance extracted (AVE) value of 0.527. The six impulsive scale items project loadings between 0.598 and 0.725, with an AVE registering 0.450. Although this AVE falls marginally below the strict 0.50 threshold, applying standards codified by Fornell and Larcker (1981), CR values residing above 0.60 conceptually satisfy adequate construct validity parameters.

**Table 3:** Reliability and convergent validity test of core psychological scales (*N* = 890).

Latent variable	Measurement items	Standardized factor loading range	Average variance extracted (AVE)	Composite reliability (CR)	Cronbach's α
Financial anxiety (Anx)	Q12–Q17	0.663–0.767	0.527	0.870	0.869
Impulsive tendency (Imp)	Q18–Q23	0.598–0.725	0.450	0.830	0.829

Discriminant validity computations confirm that the square root configurations tied to both constructs (0.726 and 0.671) structurally exceed the Pearson bivariate correlation value linking the two concepts (0.356).

Addressing limitations inherent in singular data collection methods, Harman's single-factor procedure was executed, generating a primary unrotated component representing 38.47% of aggregated variance, residing below standard alerting thresholds. While we acknowledge that the single-factor test provides only limited evidence concerning common method variance, a stringent single-factor CFA configuration produced incompatible fit metrics: χ^2^/*df* = 24.233, CFI = 0.677. Comparative testing demonstrates the two-factor proposal yields significant descriptive superiority over the singular structure [Δχ^2^(1) = 1272.394, *p* < 0.001], identifying common method variance as a non-interfering operative element within the dataset.

### 4.2 Descriptive statistics and correlation analysis

To elucidate the foundational covariation network among the focal continuous variables, a Pearson correlation matrix was computed and mapped onto a visualized heatmap (Figure 2), utilizing color gradients to index structural alignment.

**Figure 2:**
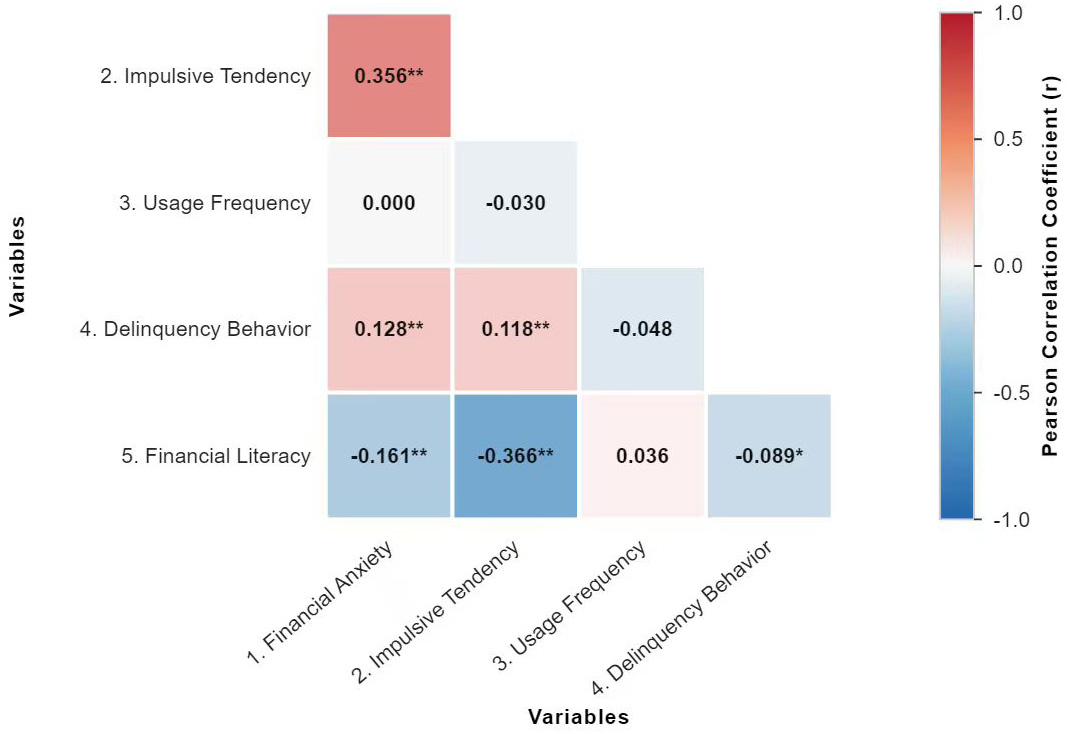
Heatmap of bivariate Pearson correlation matrix for core continuous variables. *n* = 708 (active digital credit users only). Values represent Pearson correlation coefficients (*r*). The color gradient indicates the direction and strength of the structural alignment, with red zones representing positive covariation and blue zones representing negative covariation. *p < 0.05, **p < 0.01.

As depicted in Figure 2, reported financial anxiety scores demonstrate a significant positive covariation with both impulsive buying tendency (*r* = 0.356, *p* < 0.01) and documented debt delinquency (*r* = 0.128, *p* < 0.01), mapping onto the indicated red correlation zones. Conversely, objective financial literacy exhibits a baseline negative correlation with delinquency behaviors (*r* = −0.089, *p* < 0.05, corresponding to the blue blocks). Notably, prior to controlling for confounding socio-demographic variables, the dataset presents a distinct structural characteristic: the correlation between an individual's financial anxiety level and the physical frequency of BNPL tool application fails to reach statistical significance, hovering proximate to absolute zero (*r* < 0.001, *p* = 0.995, near-white zone). This divergence indicates that the behavioral manifestation of emotional disturbance requires mapping through a more granular relational pathway rather than broad frequency metrics.

To empirically verify whether the baseline adoption of digital credit services structurally aligns with variance in psychological distress, an independent samples t-test was executed contrasting active credit users (*n* = 708) with the non-user cohort (*n* = 182). The computational output (*t* = 17.96, *p* < 0.0001) confirms a systemic asymmetry: populations engaging with BNPL platforms register mean financial anxiety scores significantly exceeding those of their non-using counterparts. Having mathematically established this uneven distribution of anxiety perception associated with tool adoption, subsequent analyses purposefully restrict focus to the active user cohort to dissect how baseline distress interacts with specific maladaptive consumption dimensions.

### 4.3 Baseline regression analysis of core traits on digital credit patterns and verification of Hypotheses H1–H3

To systematically operationalize the structural associations between emotional variables and individual credit reliance, this investigation mapped the predictive covariations linking financial anxiety to distinct consumption dimensions via specialized regression frameworks. As detailed in Table 4, within the multiple linear models predicting usage frequency (Freq) and dependence (Dep), the empirical coefficients mapped to financial anxiety returned values of 0.004 (*p* = 0.924) and 0.041 (*p* = 0.306), respectively, failing to reject the null hypothesis. This empirical observation aligns with the structural reality of contemporary digital markets: mainstream fintech interfaces ubiquitously embed frictionless payment options into rigid, daily expenditure routines (e.g., ride-hailing and food delivery). Consequently, baseline operational frequency is dictated primarily by situational necessities rather than subjective anxiety states. Given the absence of statistical significance in these direct correlational pathways, Hypothesis H1 remains unsupported.

**Table 4:** Multiple OLS regression of financial anxiety on “Buy Now, Pay Later” usage frequency and dependence (*N* = 708).

Variable	Usage frequency (Freq)		Usage dependence (Dep)	
	Model 1	Model 2	Model 3	Model 4
Financial anxiety (Anx)	–	0.004 (0.043)	–	0.041 (0.045)
Control variables (demographics)	Controlled	Controlled	Controlled	Controlled
*R* ^2^	0.005	0.005	0.005	0.007
F	1.12	0.98	1.15	1.09

Values for Financial Anxiety represent unstandardized regression coefficients (*B*) with standard errors (SE) in parentheses. All models control for gender, age, education, occupational status, and income. No coefficients for Anx reached statistical significance (*p* > 0.05).

Addressing credit delinquency patterns (Def), the analysis deployed hierarchical OLS regression to isolate the net empirical correlation attributable to subjective anxiety, independent of socio-demographic baseline variance. Within the foundational block (Block 1), the introduction of standard demographic controls yielded an initial determination coefficient (*R*^2^) of 0.0080. The subsequent integration of the financial anxiety construct (Block 2) precipitated a mathematically significant expansion in the model's explanatory capacity (*R*^2^ = 0.0242, Δ*R*^2^ = 0.0162, *p* < 0.001). The localized effect size (Cohen's *f*^2^) corresponding to this inclusion registered at 0.017. This outcome provides a critical empirical insight: holding socio-economic baselines (income and education) constant, psychological distress independently correlates with maladaptive delinquency. As indicated by the Δ*R*^2^ of 0.0162, although the practical explanatory contribution of financial anxiety within this specific step is relatively modest, its theoretical significance remains critical: it underscores that digital default extends beyond pure material constraints into the psychological domain.

As captured in Table 5, financial anxiety maintains a robust positive covariation with credit delinquency tendencies (*B* = 0.146, *p* < 0.001), indicating that perceived economic insecurity statistically parallels the deterioration of intertemporal payment discipline, thereby supporting Hypothesis H2.

**Table 5:** Multivariate regression estimates of financial anxiety on installment default and hedonic consumption preference (*N* = 708).

Variable	Delinquency behavior (Def^a^) [hierarchical linear regression (OLS)]	Hedonic consumption scenario (Scen^b^) [binary logistic regression]	OR
	***B*** **(SE)**	***B*** **(SE)**	
**Main effect independent variable**			
Financial anxiety (Anx)	0.146^***^ (0.043)	0.731^***^ (0.107)	2.078
Control variables	
Gender (Male = 1)	−0.086 (0.072)	0.216 (0.169)	1.240
Age	0.029 (0.031)	−0.061 (0.073)	0.941
Education level (Edu)	0.076 (0.043)	0.227^*^ (0.099)	1.255
Income level (Income)	0.011 (0.029)	−0.039 (0.068)	0.962
Model fit and test statistics	
Sample size (*N*)	708	708	–
Block 1 baseline model *R*^2^	0.0080	–	–
Final overall fit	Δ*R*^2^ = 0.0162^***^	Nagelkerke *R*^2^ = 0.110^***^	–
Overall model test value	Δ*F*(1,702) = 11.65^***^	Model χ^2^(5) = 58.04^***^	–

^*a*^The delinquency behavior model reports the unstandardized regression coefficients (B) of the second block (Block 2) after entering control variables and the main independent variable hierarchically (Hierarchical Entry). ^*b*^In the hedonic consumption scenario model, the dependent variable is binary (0 = necessity scenario, 1 = hedonic scenario). Values in parentheses represent the standard errors (SE). OR represents the Odds Ratio. ^*^p < 0.05, ^***^p < 0.001.

Furthermore, within the binary logistic regression framework examining consumption scenario selection, financial anxiety emerged as a significant predictor for non-essential hedonic orientations (Scen), carrying a regression coefficient of 0.731 (*p* < 0.001) and a substantial odds ratio (OR) of 2.078. This associative pattern implies an emotion-driven structural reallocation: even under conditions of perceived fiscal constraint, individuals displaying higher anxiety scores exhibit an increased likelihood of bypassing essential utility channels, instead directing credit limits toward consumption categories engineered for immediate psychological compensation or “mood repair.” This statistical skew confirming an elevated probability of hedonic misallocation among anxious cohorts functionally validates Hypothesis H3.

To visually contextualize these structural consumption shifts, a Sankey flow diagram (Figure 3) was constructed, directly mapping the allocation flow from individuals' baseline psychological states to their ultimate credit utility. The visualization solidifies the descriptive mechanics anchoring Hypothesis H3: within the low-anxiety cohort, hedonic allocations represent 55.6% of credit usage, whereas this proportionate bandwidth steeply escalates to 79.9% within the high-anxiety subdivision. This pronounced 24.3 percentage-point differential graphically captures the structural reallocation toward emotion-driven, compensatory overdrawing fueled by internalized financial insecurity.

**Figure 3:**
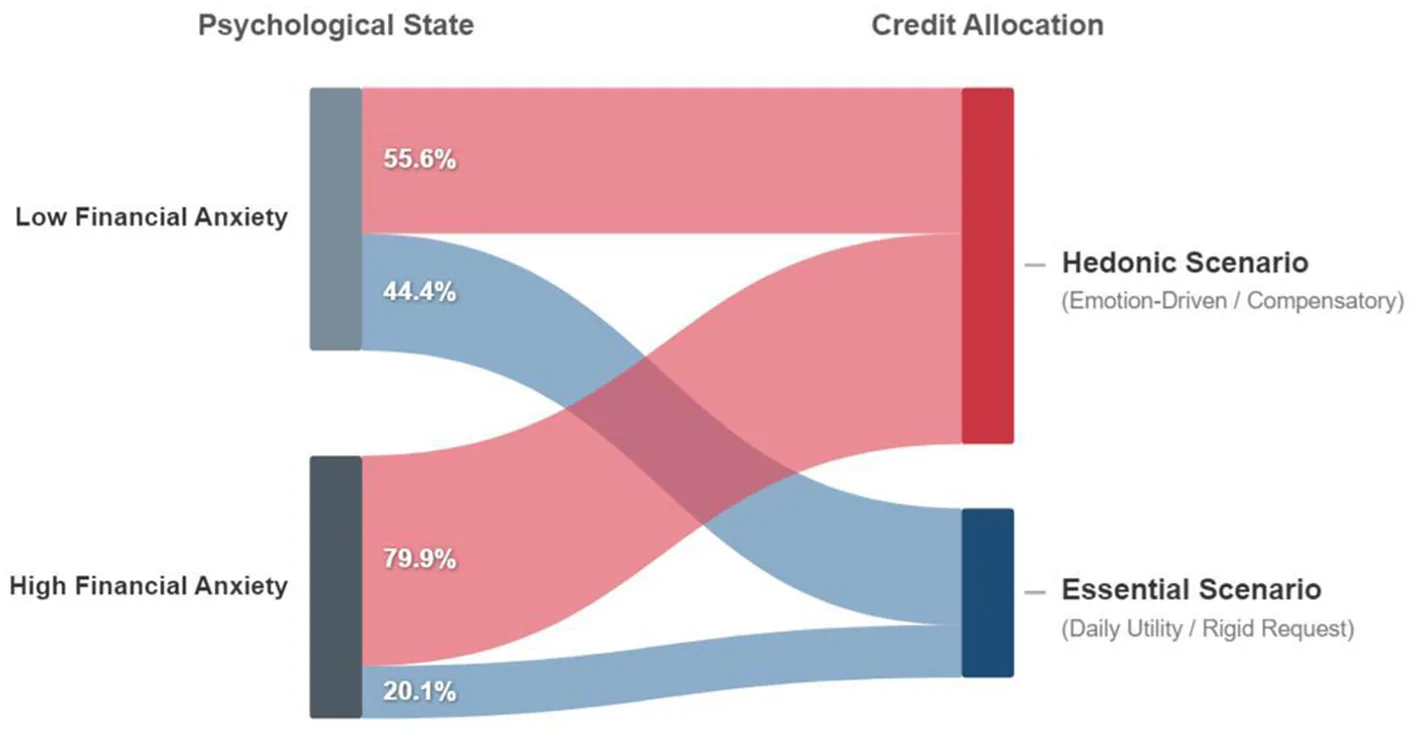
Structural reallocation of credit usage across financial anxiety groups.

Transitioning from aggregate structural shifts to individualized risk trajectories, marginal effects derived from the empirical models were mapped onto a dual-panel predictive framework (Figure 4). As demonstrated by the plotted risk curves and their accompanying 95% confidence intervals, shifting an individual's latent financial anxiety score from the baseline (1.0) toward the psychological extreme (5.0) is strongly associated with a rapid ascent in the predicted probability of initiating hedonic consumption scenarios (Figure 4A, scaling from 0.47 to 0.90). Concurrently, evaluating the continuous metric of operational debt risk, the predicted score for delinquency tendency charts a steady, upward progression from 1.62 to 2.21 (Figure 4B). By meticulously isolating these functional scales (probability vs. ordinal scores), these trajectories mathematically encapsulate the cumulative associations linking subjective economic stress to multifactorial decision-making impairments.

**Figure 4:**
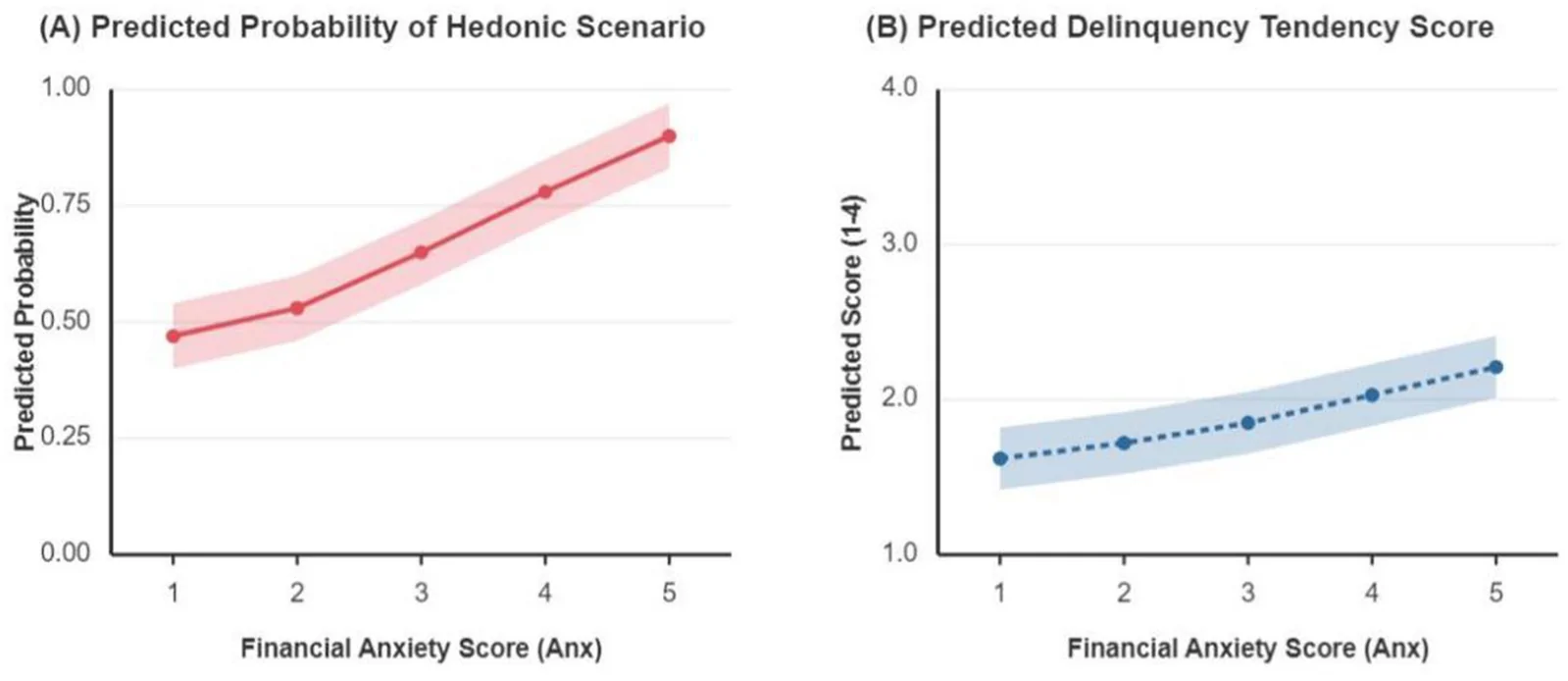
Dual-panel marginal prediction trajectories mapping the effect of financial anxiety on multifaceted credit outcomes. **(A)** Visualizes the escalating probability of non-essential hedonic consumption, while **(B)** tracks the progressive deterioration in debt delinquency scores. Shaded bands represent 95% confidence intervals.

### 4.4 Test of the mediating pathway of impulsive buying tendency and verification of Hypothesis H4

Given that emotional distress frequently co-occurs with observable behavioral deficits via constrained cognitive control (i.e., self-regulatory resource depletion), the investigation proceeded to evaluate impulsive buying tendency as a mediating variable.

Applying the conditional process framework (Hayes, 2017), this transmission mechanism was assessed within a baseline bivariate mediation model employing non-parametric Bootstrap resampling (5,000 iterations). Comprehensive path decomposition—disaggregating total, direct, and indirect effect parameters—is documented in Table 6, with the structural topology visualized in Figure 5.

**Table 6:** Path decomposition of the mediating effect of impulsive buying tendency between financial anxiety and delinquency behavior (*n* = 708).

Effect type	Path relationship	Effect size (effect)	Bootstrap standard error (boot SE)	*t*-value	*p*-value	95% boot LLCI	95% boot ULCI	Proportion of EFFECT
Total effect	Anx → Def	0.1474	0.0439	3.436	0.001	0.0582	0.2313	100.00%
Direct effect	Anx → Def (controlling for Imp)	0.1139	0.0481	2.485	0.013	0.0164	0.2076	77.27%
Indirect effect (mediation)	Anx → Imp → Def	0.0335	0.0162	–	–	0.0027	0.0666	22.73%

Boot SE, Boot LLCI, and Boot ULCI are calculated based on the non-parametric percentile Bootstrap method (5,000 resamples). Since there is no direct symmetric *t*-distribution test for indirect effects, their significance is determined solely by whether the confidence interval excludes 0.

**Figure 5:**
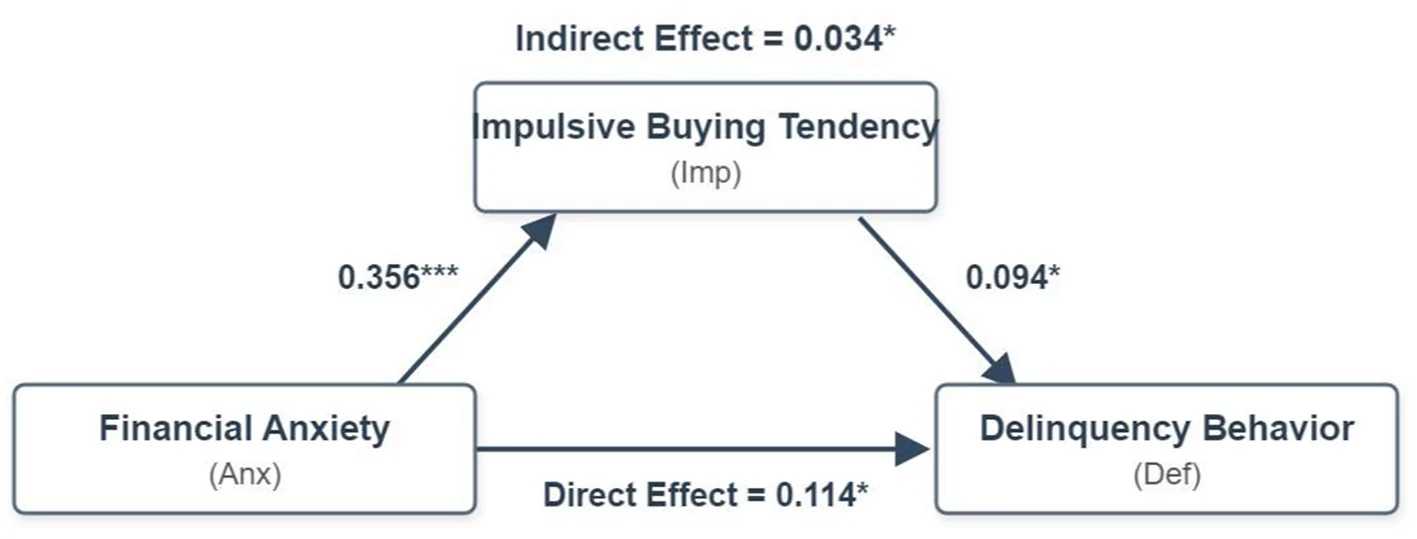
Structural path diagram mapping the mediating mechanism of impulsive buying tendency. Values presented are unstandardized path coefficients. Financial anxiety is indirectly associated with digital delinquency behavior through a pattern indicative of self-regulatory depletion, corroborating partial mediation characteristics. The significance of the indirect effect was determined using 5,000 Bootstrap resamples, where the 95% confidence interval [0.0027, 0.0666] did not contain zero. *p < 0.05, ***p < 0.001.

Specifically, the unstandardized path coefficients mapped in the model reveal that financial anxiety demonstrates a robust positive association with elevated impulsive tendency (the a path: *B* = 0.356, *p* < 0.001), which successively exhibits a significant positive covariation with delinquency behavior (the b path: *B* = 0.094, *p* < 0.05). Computational outputs verify that the indirect mediating coefficient operationalizing this correlational relay registers at 0.0335, commanding 22.73% of the total effect aggregate (0.1474). Simultaneously, the direct effect retains statistical significance (*B* = 0.1139, *p* = 0.013), governing the residual 77.27% of the correlational share. Because the 95% Bootstrap confidence intervals binding this indirect effect (LLCI = 0.0027, ULCI = 0.0666) rigorously exclude zero, the data mathematically substantiates that escalated financial anxiety is structurally accompanied by elevated trait-level impulsivity, which concurrently exhibits a robust positive covariation with subsequent default metrics. Consequently, Hypothesis H4 achieves empirical confirmation.

To mitigate cumulative measurement biases inherent in averaging multi-indicator observational scales, a supplementary Structural Equation Model (SEM) incorporating latent variable error-correction architectures was independently modeled. This SEM alignment successfully replicated the core mediation relationship, lending structural stability to the findings surrounding these psychological vulnerabilities.

### 4.5 Test of the conditional moderating mechanism of financial literacy and verification of Hypothesis H5

Having established the correlational network tethering emotional distress to impulsive behavior, this section examines whether objective cognitive resources (i.e., financial literacy) maintain the capacity to buffer these associations. Theorizing that impulsive financial behaviors might theoretically be restrained by an accurate comprehension of cumulative debt costs, objective financial literacy (FinLit) was integrated into the mediation pathway as a conditional moderator.

Evaluations utilizing multiple regression mechanics (Table 7) indicate that following the introduction of the requisite interaction protocols, the product term combining impulsive tendency and financial literacy (Imp × FinLit) failed to breach the threshold of statistical significance (*B* = −0.024, *p* = 0.611). Notably, while controlling for this interaction, the primary associative vector linking mental anxiety directly to default remained undisturbed (*B* = 0.113, *p* < 0.05). This yields a theoretically revealing contrast: while composite financial knowledge may benefit logic-based budget planning under baseline conditions, the delayed retrieval of such objective arithmetic logic proves entirely insufficient to override emotion-driven impulses once individuals are already trapped in a state of stress-induced self-regulatory depletion. Consequently, we found no evidence that objective financial literacy significantly moderated the association between impulsive buying tendency and delinquency in this sample. Given the absence of statistically significant interaction properties, Hypothesis H5 is rejected.

**Table 7:** Moderating effect test of objective financial literacy on the “impulsive trait → delinquency behavior” path.

Dependent variable: delinquency default behavior (Def)	Unstandardized coefficient (*B*)	Standard error (SE)	*t*-value	*p*-value
Constant (intercept)	1.560	0.150	10.432	< 0.001
Financial anxiety (Anx)	0.113	0.046	2.472	0.014^*^
Impulsive tendency (Imp, centered)	0.074	0.049	1.516	0.130 (not significant)
Financial literacy (FinLit, centered)	−0.050	0.041	−1.228	0.220 (not significant)
Interaction term (Imp × FinLit)	−0.024	0.047	−0.509	0.611 (not significant)

Independent variables and the moderating variable were mean-centered before entering the regression equation. The dependent variable (Def) retains its original score level. ^*^p < 0.05.

### 4.6 Robustness analysis and heterogeneity test of group characteristics

To ascertain the structural stability of the findings derived from the primary cross-section, sequential subgroup configurations and robustness protocols were executed. Crucially, addressing the methodological parameter that BNPL delinquency operates as an ordered four-category outcome, supplementary ordinal logistic regression models were executed alongside the primary OLS estimations. The substantive structural coefficients and significance directions remained fully intact. This confirms that the positive covariation connecting financial anxiety to delinquency, as well as the indirect mediating path, did not artifactually result from continuous scale specifications, thereby maximizing methodological credibility.

Initially addressing the premise that margin-constrained demographics exhibit heightened environmental vulnerability, a targeted sub-sample comprising respondents generating monthly revenues below 5,000 RMB (*n* = 332) was isolated for parallel estimation (Table 8).

**Table 8:** Robustness re-estimation test of key paths under income grouping.

Alternative path scheme for testing	Anx → Def prediction coefficient	Interaction product term moderating coefficient	Conclusion consistency
Restricted sample group (monthly income < 5,000, *n* = 332)	0.077 (*p* = 0.224)	−0.036 (*p* = 0.572)	Moderating effect is not significant (consistent with baseline)

Within this restricted testing parameter, the direct correlational linkage between financial anxiety and delinquency status exhibits statistical attenuation (*B* = 0.077, *p* = 0.224). This divergence theoretically suggests that for socio-economically marginalized cohorts navigating absolute liquidity constraints, digital delinquency is predominantly tethered to the physical absence of capital, thereby compressing the observable variance strictly attributable to subjective psychological anxiety. However, testing the interactive properties within this sub-sample reaffirms the baseline architecture: the interaction term (Impulse × FinLit) remains definitively non-significant (*B* = −0.036, *p* > 0.10). This uniformity corroborates the prior deduction that objective financial awareness fails to provide a functional cognitive buffer against trait-driven delinquency, solidly confirming boundary condition limitations across diverse economic brackets.

Further addressing demographic heterogeneity, an Analysis of Variance (ANOVA) was deployed to track potential systematic variances in the absolute means of the central psychological and consumption metrics across distinct age and educational classifications. Output metrics documented that partial eta squared values (Partial η^2^) spanning these demographic variables remained sequestered within an exceptionally restricted effect interval (0.0001 to 0.006), lacking main effect significance (*p* > 0.10). This homogeneity structurally infers that generalized anxiety perceptions and overlapping debt behaviors permeate equally across stratified social classifications without severe local polarization.

Finally, evaluating the configurational invariance of the core covariation matrix (Financial Anxiety → Impulsive Tendency → Delinquency Performance), the methodology mobilized a Multi-Group Structural Equation Model (Multi-Group SEM). Segregating the dominant user populations into student collectives (*n*^1^ = 165) and actively employed cohorts (*n*^2^ = 400), path equivalence constraint tests were administered. The nested comparative analysis generated negligible fit deterioration against the unconstrained equivalent [Likelihood ratio test Δχ^2^(2) = 1.64, *p* = 0.440]. This structural symmetry indicates that irrespective of differing cash flow stabilities, the maladaptive triad binding perceived insecurity, behavioral impulsivity, and structural default demonstrates a highly convergent psychological functionality across varying social roles.

## 5 Discussion

### 5.1 Asymmetric covariation and emotion-driven compensatory consumption

Anchored within the classical economic parameters of life-cycle theory, rational agents confronting financial stress or anticipated income volatility theoretically activate spontaneous defensive mechanisms, typically manifested through the contraction of elastic spending and the accumulation of precautionary reserves. The current cross-sectional dataset partially reflects this defensive orientation: generalized financial anxiety fails to positively predict the overarching frequency or aggregate bill dependence of BNPL utilization. Confronting perceived fiscal constraints, respondents generally maintain baseline restraint regarding categorical debt exposure.

This overarching frequency restraint, however, does not equate to the mitigation of covert digital credit risks. The bivariate analysis and multivariate regression models mapped within this study isolate an “asymmetric” covariation topology: while the active credit cohort registers significantly elevated baseline financial anxiety compared to non-users, variance in this anxiety among active users does not statistically govern physical transaction frequency. Instead, it demonstrates a robust positive covariation with non-essential hedonic allocations and structural delinquency tendencies.

This divergence signifies that psychological distress bypasses generalized utility patterns, manifesting instead through structural consumption skew and the erosion of intertemporal repayment perimeters. Evaluated through the psychological lens of Compensatory Consumption Theory (Rucker and Galinsky, 2008), persistent financial anxiety functions not merely as a perceived deficit in monetary liquidity, but as a severe degradation of an individual's locus of psychological control. Consequently, leveraging digital credit to acquire hedonic goods exceeding immediate budget constraints transcends simple material acquisition; it operates as an emotion-driven compensatory maneuver engineered to temporarily alleviate negative affect and restore psychological homeostasis.

Within this behavioral architecture, the frictionless affordances of mobile credit interfaces serve as critical environmental catalysts (Hung et al., 2025). Routine digital checkout topographies featuring “default credit initiation” are highly predisposed to interact with impulsive compliance when users navigate stress-induced self-regulatory depletion, effectively deferring psychological repayment pressure at the execution locus. As confirmed by the pathway analyses, affective volatility strongly coincides with escalating impulsive traits. By bypassing the traditional “pain of paying,” frictionless algorithms seamlessly accommodate the high co-occurrence of these inhibitory deficits. Ultimately, while this compensatory pathway facilitates temporary affective repair, it is paradoxically accompanied by the covert accumulation of localized debt risks (Nusir et al., 2026).

### 5.2 The bounds of cognitive rationality and the case for emotion-focused resilience

A profound theoretical extension of these empirical profiles emerges from the non-significant moderating capacity of objective financial literacy. Traditional behavioral finance paradigms postulate that cognitive knowledge stocks should fundamentally buffer individuals against debt exposure. Contrastingly, the empirical output dictates that purely cognitive resources (e.g., compound interest calculation and rational risk assessment) fail to significantly disrupt the covariation linking acute impulsive traits with actualized delinquency defaults.

Interpreted through dual-process psychological frameworks, this absent moderation is theoretically revealing: when subjects remain entrenched within states of ego depletion precipitated by chronic economic distress, their executive rational pathways (“System 2” processing) are severely compromised. At the highly affective apex of an impulsive checkout decision, the cognitive retrieval of abstract mathematical logic lacks the necessary momentum to override emotion-driven impulses. This dynamic dictates that addressing digital delinquency necessitates interventions extending strictly beyond cognitive financial education. Because explicit cognitive retrieval lacks momentum during highly affective checkouts, intervention frameworks mapping onto this theoretical depletion mechanism must prioritize psychological resilience over mere mathematical proficiency. Ultimately, addressing this emotional dysregulation offers a viable mechanism to fracture the cyclical pathological loop: Financial Stress → Self-Criticism → Self-Regulation Failure (Impulsive BNPL Usage) → Credit Delinquency.

### 5.3 Cross-cultural examination of digital ecosystems and cognitive vulnerabilities

Projecting these localized micro-mechanisms onto the macro-structural canvas of consumer finance facilitates a broader assessment of digital credit behaviors and their institutional contingencies. Juxtaposing the current dataset against precursor studies rooted in North American contexts exposes distinct hierarchical credit topographies. In developmental market ecosystems, digital credit access typically correlates with broad expansions in unified retail spending. Conversely, within the mature, highly integrated domestic digital environment investigated here, generalized frequency correlations dissipate, yielding to the pronounced exposure of structural risks, particularly intertemporal delinquency vulnerabilities. This structural transition indicates that assessing total market expansion is no longer sufficient; academic inquiry must pivot to analyze how localized fintech integrations restructure usage patterns and heavily burden cognitive control requirements.

The heterogeneity characterizing behavior across diverse financial architectures warrants specific scrutiny. Contrasting with the gradual, linear evolution of Western credit cultures, domestic consumer finance exhibits distinctive “leapfrog” infiltration characteristics. Emerging consumer cohorts are routinely submerged in high-frequency, interactive mobile credit environments prior to experiencing the formalized “domestication” boundaries of traditional credit card algorithms (Izham et al., 2025; Podin et al., 2025). Simultaneously, dominant “Super Apps,” which synthesize social networking with entertainment, seamlessly embed financial conduits directly into daily social interactions, systematically stripping away the physical and spatial decision-making buffers historically afforded to consumers.

This systemic elimination of environmental friction places unparalleled, sustained demands on individual self-regulation capabilities. Statistically, this environmental reality aligns with the identified steep covariation connecting impulsive traits to structural default. Validating prior assertions regarding BNPL's capacity to magnify financial vulnerability (Gerrans et al., 2022), the multi-group invariance tests within this study confirm that the structural pathway coupling subjective anxiety and impulsivity to delinquency generalizes evenly across stratified class demographics (e.g., student vs. employed vectors). Ultimately, while the highly integrated domestic digital ecosystem epitomizes transactional inclusivity, its environmental affordances—specifically frictionless interfaces—are intrinsically coupled with severe psychological hurdles, empirically manifesting as an accelerated covariation slope linking unmitigated subjective stress to structural credit failure.

## 6 Conclusions, practical implications, and future outlook

### 6.1 Conclusions

Utilizing a cross-sectional framework analyzing 708 active digital credit users, this investigation maps the psychological architecture connecting perceived financial anxiety to maladaptive credit decision-making. Accounting for demographic baseline variance, the network configuration reveals that while persistent financial anxiety lacks structural correlation with generalized usage frequency, it serves as a robust statistical predictor for both hedonic consumption skew and severe delinquency risks. Specifically, subjective economic distress is indirectly tethered to structural default via the amplification of impulsive buying traits, which function as behavioral proxies for self-regulatory deficits.

Crucially, objective financial literacy fails to conditionally moderate this pathway. This outcome establishes that when individuals operate under psychological distress and sequential ego depletion, the buffering utility of rational cognitive reserves is precipitously compromised. Relying exclusively on factual financial knowledge proves inadequate for hedging against emotion-driven irrationality within highly integrated fintech environments. This affect-driven maladaptive trajectory demonstrates structural invariance across divergent socio-economic profiles, underscoring that baseline liquidity and occupational standing do not intrinsically confer psychological immunity against digital debt traps.

### 6.2 Practical and interventional implications

Guided by these empirical insights, this study pivots beyond conventional economic literacy paradigms, proposing protective frameworks grounded in choice architecture and targeted psychological intervention to foster digital financial wellbeing.

From a regulatory and platform-design perspective, fintech ecosystems must transition from optimizing frictionless extraction toward cultivating psychologically informed financial health. Incorporating strategically deployed “behavioral friction” is essential to interrupt the momentum of acute impulsive reactivity. For instance, platforms should dismantle “default opt-in” mechanisms for non-essential hedonic categories, requiring instead manual, deliberate confirmation. Furthermore, algorithmic identification of rapid debt accumulation—particularly during cognitively depleted periods such as late-night hours—should trigger automated “limit freezes” and “cooling-off intervals.” These choice-architecture modifications restore the temporal and spatial bounds necessary for depleted users to recalibrate executive control.

Concurrently, public support and educational systems must acknowledge the limits of purely rational processing. Because traditional pedagogies emphasizing compound interest logic exhibit minimal efficacy against affect-driven default, financial education must integrate principles of Financial Therapy and psychological empowerment. Incorporating Cognitive Behavioral Therapy (CBT) modules and emotion-regulation training can equip individuals to accurately identify stress-induced triggers. Critically, fostering self-compassion frameworks allows users to process monetary setbacks independently of destructive, threat-inducing self-criticism. Establishing this psychological resilience acts as a cognitive shield against environmental digital inducements, fundamentally rehabilitating the individual's capacity for sustained, rational intertemporal planning (Schomburgk and Hoffmann, 2023).

### 6.3 Limitations and future research directions

The conceptual parameters of these findings are necessarily bounded by the methodological design. Anchored in a single-interval cross-sectional survey, the empirical outputs isolate statistical covariation rather than definitive causal sequences, leaving the theoretical model susceptible to reverse causality. While our framework structures financial anxiety as the antecedent to digital delinquency, the inverse trajectory—where unmanageable BNPL debt accumulation, aggressive collection practices, and ensuing late penalties directly precipitate extreme psychological distress—is equally plausible and represents a pervasive real-world feedback loop. Consequently, strong causal claims must be interpreted with caution. Additionally, despite controlling for key socio-demographic baselines, the estimations remain vulnerable to Omitted Variable Bias (OVB). Unobserved heterogeneous factors—such as underlying clinical mental health conditions (e.g., undiagnosed depression), variations in early childhood financial socialization, or sudden exogenous macroeconomic shocks (e.g., sudden medical expenses)—could simultaneously drive both heightened anxiety and credit default, confounding the isolated net effects.

A critical theoretical limitation concerns the unobserved mediating mechanism. The current design theoretically infers a process of self-regulatory resource consumption but does not directly measure state-level ego depletion, relying instead on trait-level impulsive buying tendency. Finally, measurement constraints must be explicitly acknowledged. While maintaining established validity, the reliance on self-reported psychometric scales introduces inherent subjective reporting variance when utilized as proxies for physical default behaviors. Furthermore, objective financial literacy was operationalized utilizing a concise three-item metric. Although established as a baseline economic proxy, a three-item scale inherently possesses limited construct coverage. It captures fundamental mathematical rationality but may not fully encapsulate the nuanced digital financial literacy and debt literacy specifically required within contemporary BNPL ecosystems.

To transcend these empirical confines, future trajectories must prioritize methodological diversification. Beyond aggregating longitudinal panel data to untangle temporal precedence via Cross-Lagged Panel Models (CLPM), future research must endeavor to measure self-regulatory depletion directly. Investigators should adopt Ecological Momentary Assessment (EMA) technologies via mobile interfaces. Capturing real-time psychophysiological fluctuations in ego depletion and affective states exactly at the point of digital checkout would yield unparalleled ecological validity. Synthesizing such localized field data with exogenously manipulated laboratory stress paradigms will ultimately complete the explanatory matrix regarding human self-regulation within digital consumer credit ecosystems.

## Data Availability

The original contributions presented in the study are included in the article/Supplementary material, further inquiries can be directed to the corresponding author.
