# Supplementary material for: The psychology of digital credit: the association of financial anxiety and impulsive traits with “buy now, pay later” delinquency
Source: Front Psychol. 2026 Oct 12;17:1950938. doi: 10.3389/fpsyg.2026.1950938 (PMC13696469; doi:10.3389/fpsyg.2026.1950938)
Supplement: Supplementary file 1 [file Data_Sheet_1.zip › Supplementary_Material_Survey_and_Codebook(2).docx]

Survey Questionnaire and Variable Codebook

**Part I: Original Survey Questionnaire**

“Cross-Sectional Survey Questionnaire on Consumption Behavior and Financial Feelings”

**Section 1: Preamble and Informed Consent**

Dear respondents:

Hello! We are an academic team conducting research on consumption behavior and physical psychology. This questionnaire aims to understand current public daily consumption habits and their accompanying psychological states.

Your answers are crucial to this study. The questionnaire is completely anonymous, and the collected data only contain statistical patterns, never involving personal privacy, and are used solely for academic research. Please feel free to fill it out based on your actual situation. The entire process takes approximately 6-8 minutes.

Q1. Are you at least 18 years old and do you agree to participate in this anonymous academic survey? (Screening Question)

A. Yes, I am at least 18 years old and agree to participate (Continue the questionnaire)

B. No (Terminate the questionnaire)

**Section 2: Basic Information (Demographic Characteristics)**

Q2. Your gender is:

A. Male

B. Female

C. Prefer not to disclose

Q3. Your age group is:

A. 18-25 years old

B. 26-30 years old

C. 31-40 years old

D. 41-50 years old

E. Above 50 years old

Q4. Your highest education level (including currently enrolled) is:

A. High school/technical school and below

B. Junior college/vocational college

C. Undergraduate

D. Master’s and above

Q5. Your current occupational status is:

A. Full-time student

B. Full-time job (enterprise employee, civil servant, public institutions, etc.)

C. Freelance/flexible employment/self-employed

D. Unemployed/out of work

Q6. Your current average monthly disposable income after tax (if you are a student, please calculate based on monthly living expenses and part-time income) is approximately:

A. Below 3,000 RMB

B. 3,000 - 5,000 RMB

C. 5,001 - 10,000 RMB

D. 10,001 - 20,000 RMB

E. Above 20,000 RMB

**Section 3: “Buy Now, Pay Later” Usage Patterns (Dependent Variables)**

The “Buy Now, Pay Later” products in this questionnaire mainly refer to digital credit methods with credit advance and deferred repayment functions, such as Huabei, JD Baitiao, WeChat Fenfuyu, credit card overdraft installments, and installment purchases on various e-commerce platforms.

Q7. In the past 12 months, have you used the above-mentioned “Buy Now, Pay Later” products for consumption? (Key Screening Question)

A. Yes (Please continue to answer Q8)

B. No (Please skip directly to Section 4 and start answering from Q12)

Q8. [Usage Frequency] The frequency with which you usually use “Buy Now, Pay Later” products is approximately:

A. Almost every day

B. Several times a week

C. Several times a month

D. Rarely used (only once every few months)

Q9. [Usage Dependence] What is the approximate proportion of your monthly “Buy Now, Pay Later” bill to your total monthly expenditure?

A. Below 10% (occasional use, no dependence)

B. 10% - 30% (assisting daily consumption)

C. 31% - 50% (heavy use)

D. Above 50% (highly dependent on this type of credit)

Q10. [Consumption Scenarios] In which types of consumption do you mainly tend to use “Buy Now, Pay Later”? (Multiple choices allowed)

A. Daily necessities (groceries, dining expenses, utility bills)

B. Electronic products and digital technology (such as buying mobile phones, computers, etc.)

C. Clothing, shoes, bags, and cosmetics

D. Leisure, entertainment, and travel (such as concerts, booking hotels and flights, etc.)

E. Game top-ups, live stream tipping, or virtual services

Q11. [Delinquency and Debt Behavior] In the past 12 months, what has been your primary repayment status on “Buy Now, Pay Later” products?

A. Never overdue, always paid in full and on time before the bill generation or on the repayment date

B. Experienced short-term delinquency due to forgetfulness, but paid in full within a few days

C. Frequently chose “minimum payment” or voluntarily opted for “bill installment” to defer repayment due to tight funds

D. Experienced multiple delinquencies due to fund shortages, resulting in actual late payment fees/penalties

**Section 4: Financial Anxiety Scale**

Please recall your actual inner feelings in the past six months and rate the following statements. 1 = Completely disagree, 2 = Moderately disagree, 3 = Neutral, 4 = Moderately agree, 5 = Completely agree.

Q12. Just thinking about my personal financial situation or account balance makes me feel anxious and uneasy. [1] [2] [3] [4] [5]

Q13. When I need to pay bills or when the monthly repayment date approaches, I feel significant psychological stress. [1] [2] [3] [4] [5]

Q14. I have had trouble sleeping because of worries about my money, expenses, or debt. [1] [2] [3] [4] [5]

Q15. I often worry that I will run out of money before my next paycheck (or receiving living allowance). [1] [2] [3] [4] [5]

Q16. Faced with possible unexpected necessary expenses in the future (such as medical treatment, car repair, job loss, etc.), I feel extremely insecure. [1] [2] [3] [4] [5]

Q17. I often feel that I am gradually losing control over my spending. [1] [2] [3] [4] [5]

**Section 5: Impulsive Buying Tendency Scale**

Please rate the degree of agreement with the following statements based on your actual daily consumer shopping habits. 1 = Completely disagree, 2 = Moderately disagree, 3 = Neutral, 4 = Moderately agree, 5 = Completely agree.

Q18. I often buy things spontaneously without any prior planning. [1] [2] [3] [4] [5]

Q19. It is hard for me to control my impulse to buy whenever I see something I like while window shopping or browsing online. [1] [2] [3] [4] [5]

Q20. The phrase “buy now, think about it later” perfectly describes my consumption characteristics. [1] [2] [3] [4] [5]

Q21. Even if I don’t have enough cash temporarily, I want to own a desired product immediately when I see it. [1] [2] [3] [4] [5]

Q22. Sometimes I buy things just because the shopping experience feels good at the time, not because I really need it. [1] [2] [3] [4] [5]

Q23. Once I want to buy a product, I cannot resist paying for it immediately, even if it is clearly beyond my daily budget. [1] [2] [3] [4] [5]

**Section 6: Objective Financial Literacy Test**

The following three questions are used to test the public’s basic financial knowledge. Please make choices based on your first judgment. If you are really not sure, please choose “I don’t know” directly. Do not consult any resources.

Q24. Suppose you have 100 RMB in a savings account with an annual interest rate of 2%. If you leave the money untouched, how much will you have in your account after five years?

A. More than 102 RMB

B. Exactly 102 RMB

C. Less than 102 RMB

D. I don’t know

Q25. Suppose the interest rate on your savings account is 1% per year, and the current inflation rate is 2% per year. Using only the money in this account, the actual goods you can buy after one year compared to today will be:

A. More than today

B. The same as today

C. Less than today

D. I don’t know

Q26. Please judge whether the following statement is true or false: “Generally speaking, buying a single company’s stock has lower investment risk for an average person than buying an equity fund (index fund) that contains multiple stocks.”

A. True

B. False

C. I don’t know

Thank you very much for taking time out of your busy schedule to participate in this survey! Wish you a happy life!

#### **Part II: Variable Assignment and Analytical Codebook**

For the 890 collected questionnaires (among which 708 credit users with Q7 = ‘A’ were filtered out for the regression and mediation analysis models), the variable definitions and digital mapping logic in the statistical software are specified as shown in the table below:

| Empirical Symbol | Questionnaire Item Source | Variable Definition and Calculation Method | Software Input Coding and Numerical Mapping Rule |
| --- | --- | --- | --- |
| Anx (Financial Anxiety) | Q12 - Q17 | Arithmetic mean of the 6 item scores of the FAS scale | Mean values after directly summing the single item scores from 1 (completely disagree) to 5 (completely agree), resulting in a continuous variable between 1.00 and 5.00. |
| Imp (Impulsive Trait) | Q18 - Q23 | Arithmetic mean of the 6 item scores of the IBTS scale | Mean values after directly summing the single item scores from 1 (completely disagree) to 5 (completely agree), resulting in a continuous variable between 1.00 and 5.00. |
| Freq (Usage Frequency) | Q8 | Self-reported cyclical usage frequency | [Reverse scoring conversion performed so that score increase represents frequency rise] Original options mapped as: (D) Rarely used → 1; (C) Several times a month → 2; (B) Several times a week → 3; (A) Almost every day → 4. |
| Dep (Dependence) | Q9 | Proportion of bill amount to current total personal expenditure | Original options: (A) Below 10% → 1; (B) 10%-30% → 2; (C) 31%-50% → 3; (D) Above 50% → 4. |
| Scen (Consumption Scenario) | Q10 | Classification of credit installment consumption scenarios | Dichotomous variable: - If only A (daily necessities) is selected in the multiple-choice question → 0 (essential expenditure scenario); - If any one or more of B, C, D, and E are checked → 1 (non-essential hedonic scenario). |
| Def (Delinquency Behavior) | Q11 | Repayment status of payment in the past 12 months | Original options: (A) Never overdue → 1; (B) Delayed payment for a few days due to forgetting SMS → 2; (C) Experienced minimum payment or voluntarily opted for installment extension → 3; (D) Multiple substantive credit defaults resulting in late fees → 4. |
| FinLit (Financial Literacy) | Q24 - Q26 | Number of correct answers to concepts regarding financial management, inflation, and diversification | Objective scoring for three questions [Answer Key: Q24 = A, Q25 = C, Q26 = B] (correct answer scored as 1, incorrect answer or choosing “I don’t know” scored as 0), summed to obtain a score between 0 and 3. |
